# Age-Dependent Correlation between Immune Parameters and Symptoms in Young Adults with Schizophrenia

**DOI:** 10.2174/1570159X23666250407092539

**Published:** 2025-04-07

**Authors:** Cheng Zhu, Xiaoni Guan, Nan Chen, Meihong Xiu, Yuanyuan Huang

**Affiliations:** 1 School of Mental Health, Zhejiang Provincial Clinical Research Center for Mental Disorders, The Affiliated Wenzhou Kangning Hospital, Wenzhou Medical University, Wenzhou, China;; 2 Peking University HuiLongGuan Clinical Medical School, Beijing HuiLongGuan Hospital, Beijing, China;; 3 Department of Psychiatry, The Affiliated Brain Hospital, Guangzhou Medical University, Guangzhou, China;; 4 Guangdong Engineering Technology Research Center for Translational Medicine of Mental Disorders, Guangzhou, China;; 5 Key Laboratory of Neurogenetics and Channelopathies of Guangdong Province and the Ministry of Education of China, Guangzhou Medical University, Guangzhou, China

**Keywords:** Schizophrenia, age, neutrophil, lymphocyte, NLR, young adult

## Abstract

**Background:**

Schizophrenia (SCZ) is a debilitating mental disorder of unknown etiology. It is characterized by both positive and negative symptoms. Increasing evidence reports the role of immune abnormalities in the pathogenesis of SCZ.

**Objective:**

This study aimed to examine the potential effect of age on the associations between clinical symptoms and immune parameters in drug-naïve first episode SCZ (DNFES).

**Methods:**

A total of 64 young DNFES patients were recruited and divided into younger and older groups according to the median age. Immune parameters, including neutrophil (NEU), lymphocyte (LYM), and neutrophil-to-lymphocyte ratio (NLR) values, were measured and compared between the younger and older patients to investigate the potential effect of age on the correlations between immune parameters and clinical symptoms.

**Results:**

We found that NEU and NLR were significantly higher in patients compared to healthy controls, with even higher values observed in the younger group than in the older group. In addition, NEU count was correlated with clinical symptoms in older patients, while NLR was correlated with symptoms in younger patients. Linear regression analysis showed that NEU or NLR values were associated with the clinical symptoms in patients with SCZ after controlling confounders.

**Conclusion:**

Our study suggests that young adult patients had abnormal immune parameters. Furthermore, age mediated the relationships between immune parameters and symptom severity. This study provides further evidence that abnormal immune parameters, particularly an increased innate immune response, may be involved in the pathophysiology of SCZ.

## 1 INTRODUCTION

Schizophrenia (SCZ) is a chronic, disabling mental illness that usually occurs in early adulthood. The clinical symptoms of SCZ are generally classified into positive symptoms, negative symptoms, and cognitive deficits. Patients suffering from SCZ usually exhibit one or more clinical symptoms [1]. The etiology of SCZ is not fully understood. It is widely accepted that SCZ is caused by a complex interplay of genetic and environmental factors [2, 3], especially during critical periods of neurodevelopment, such as late adolescence or early adulthood. Clinical evidence also confirms that the incidence of SCZ peaks at an average age of 25 years, with another peak occurring after 40 years [4, 5]. In this study, we focused on young adult patients with SCZ between 18 and 30 years to examine their disease characteristics.

There is growing evidence that immune dysfunction and low-grade neuroinflammation are involved in the development and progression of SCZ [6, 7]. Changes in the immune system have been observed in individuals with SCZ [8, 9], such as changes in the number and function of immune cells, as well as in the levels of cytokines and other immune-related molecules. Evidence from postmortem brain studies and clinical investigations of SCZ patients suggests alterations in immune system genes and abnormal levels of immune-related components [10-12]. Neuroinflammation caused by immune activation may affect brain development and function, leading to changes in brain regions associated with this disorder. Low-grade inflammation might affect synaptic plasticity, neuronal connectivity, and neurotransmission, all of which are implicated in SCZ. In particular, epidemiological and animal studies have linked maternal infection during pregnancy to an increased risk of SCZ in offspring [13-15]. These findings suggest that immune dysregulation may be a key feature of SCZ.

Total white blood cell (WBC) count is a marker of low-grade inflammation. It is one of the most common standard laboratory tests in patients with psychiatric disorders. WBC has been found to correlate with SCZ in several studies [16, 17]. A meta-analysis of total and differential leukocyte counts in SCZ revealed that total WBC, monocytes, and neutrophil (NEU) counts were significantly higher in individuals with SCZ compared to healthy controls [17]. More recently, it has been shown that NEU, lymphocyte (LYM) counts, and NEU to LYM ratio (NLR) are higher in SCZ than in healthy controls [18-21]. Evidence supports the link between peripheral inflammation and central nervous system (CNS) inflammation. Numerous neuropathological and neuroimaging studies have reported that microglia activation and subsequent proinflammatory cytokine production may disrupt the blood-brain barrier, especially in neurologic disorders with comorbid psychiatric symptoms [22]. Indeed, in some patients with higher pro-inflammatory cytokines, elevated blood-derived immune-related molecules, rather than that inherent to the brain, were observed in SCZ [23].

Identifying immune-related molecules that are altered in SCZ may lead to the development of biomarkers that can help diagnose the disorder, monitor disease progression, and develop new therapeutic strategies. Although evidence supports the involvement of immune system disturbance in SCZ, there are inconsistencies among these studies [24-27], which may be related to the confounding effect of including patients of different ages within the studies. However, previous studies have not fully focused on monitoring the potential effect of age. The current study investigated the relationship between immune parameters and symptoms in the first episode and focused particularly on the early stage of SCZ. Only young adult patients with SCZ were recruited to explore the relationship of immune molecules with clinical symptoms in the initial phase of SCZ and to clarify earlier results that were confounded by a heterogeneous population with respect to age.

In this study, we analyzed the differences in immune parameters, such as NEU, LYM, and NLR values, between young adult patients with SCZ and healthy controls and further explored the different relationships between immune parameters and the severity of clinical symptoms in the younger and older groups of patients. This study aimed to demonstrate 1) whether there were significant differences in immune parameters between patients and controls or between the younger and older groups and 2) whether age had an impact on the relationship between immune parameters and symptoms of SCZ.

## 2 METHODS

### 2.1 Patients

Sixty-four young, adult drug-naïve first-episode SCZ (DNFES) patients were recruited from three psychiatric hospitals. Three experienced psychiatrists diagnosed SCZ according to the Diagnostic and Statistical Manual of Mental Disorders, Fourth Edition (DSM-IV). All patients should meet the following inclusion criteria: 1) 18 to 30 years of age; 2) duration of illness not exceeding 3 years; 3) first episode; and 4) drug-naïve or received < 2 weeks of antipsychotic medications. Exclusion criteria included: 1) presence of major physical disorders; 2) a history of allergy or autoimmune disease or use of anti-immune agents; 3) met any other major Axis I disorder; and 4) pregnant and breastfeeding females. Of all 64 patients, 39 were drug-free, and 25 were treated previously with antipsychotics for less than 2 weeks.

Fifty-four age- and sex-matched healthy controls were recruited through advertisements. To ensure that healthy subjects or their first-degree relatives had not experienced a lifetime Axis-I mental illness, the participants were asked to undergo an interview to screen for psychiatric/medical history, which was conducted by an experienced psychiatrist. The screening procedure included the non-patient edition of the Structured Clinical Interview for DSM Disorders (SCID). The protocol was approved by the Ethics Committee of Beijing Huilongguan Hospital. Prior to enrollment, all participants signed an informed consent form.

### 2.2 Clinical Interview

The Positive and Negative Syndrome Scale (PANSS) was used to assess the severity of clinical symptoms. The inter-rater correlation coefficient for the PANSS score was 0.94. Sociodemographic data, including sex, onset age, and body mass index (BMI), were collected using a detailed questionnaire.

We divided the patients into two groups according to median age: from 18 to 21 years old as the younger group and ≥ 22 as the older group.

### 2.3 Laboratory Measurements

Five milliliters of blood were extracted from patients in vacuum tubes between 7:00 am and 8:00 am. WBC parameters were evaluated using a SYSMEX XN-3000 assembly line according to the manufacturer’s instructions in the hospitals.

### 2.4 Statistical Analyses

SPSS for Windows, version 20.0, was used for the statistical analysis. The significance threshold was set at *p* < 0.05.

The demographic and clinical variables and total WBC count were compared between patients and controls, as well as between the younger and older groups. In our study, the continuous variable was expressed as the mean ± standard deviation (SD) and compared using the analysis of variance (ANOVA). Categorical data were reported as n (%) and compared using the *X*^2^. Pearson correlation analysis was carried out between demographic and clinical variables, as well as total WBC in the younger and older patients, respectively. Linear regression analysis was performed to examine the associations between immune parameters and the severity of clinical symptoms in both groups. In the regression model, severity of symptoms was entered as the dependent variable, and onset age, sex, BMI, and immune parameters were added as independent variables.

## 3 RESULTS

Patients with SCZ (male/female: 35/29) had higher NEU and NLR values than healthy controls (male/female: 34/20) (for NEU: 4.6 ± 1.6 *vs.* 3.9 ± 1.3, F=4.2, *p =* 0.04; for NLR: 2.3 ± 1.4 *vs.* 1.8 ± 0.8, F=5.3, *p =* 0.02).

Of all 64 patients recruited, 29 were in the younger group, and 35 were in the older group. Older patients had higher NEU and NLR values than younger patients (F=5.1, *p =* 0.03; F=4.0, *p =* 0.05) (Table **1**). After controlling for sex, educational level, and BMI, the difference in NEU count between the two groups remained significant (*p <* 0.05).

In the younger group, we found positive correlations between NEU count PANSS total scores (r=0.38, *p =* 0.04) and between NLR and general psychopathology (r=0.39, *p =* 0.04) and the total score (r=0.45, *p =* 0.01) (Table **2**). After Bonferroni corrections (*p =* 0.05/4=0.0125), the correlations between NLR values and PANSS total score remained significant (p_Bonferroni_<0.05). However, the associations between immune parameters and clinical symptoms did not pass the more conservative Bonferroni multiple comparison-corrected thresholds of *p <* 0.0125 (two-tailed).

In the older patients, we found NEU count and NRL values were positively associated with negative symptoms (NEU: r=0.51, *p =* 0.002; NLR: r=0.36, *p =* 0.03), and the PANSS total scores (NEU: r=0.44, *p =* 0.007; NLR: r=0.35, *p =* 0.04). After Bonferroni corrections, the associations between NEU and negative symptoms or PANSS total score remained significant (p_Bonferroni_<0.05). However, there was no significant correlation between LYM count and clinical symptoms (all *p* > 0.05).

To minimize the potential impact of confounders, linear regression analysis was performed separately for younger and older patients. We found that in the older group, NEU count was associated with negative symptoms (β=0.390, t=2.152, *p =* 0.040, *R*^2^=0.286) and PANSS total score (β=0.489, t=2.617, *p =* 0.014, *R*^2^=0.240) after controlling for age of onset age, sex, and BMI. In the younger group, we found that NLR values were positively associated with PANSS total score after controlling for these confounders (β=0.537, t=3.305, *p =* 0.003, *R*^2^=0.467).

## 4 DISCUSSION

A limited number of studies have examined the differences in immune parameters between young adult patients with SCZ and healthy controls. This study revealed that young DNFES patients had significantly higher NEU and NLR values than healthy controls. When the patients were divided into younger and older groups, we found that older patients had even higher NEU and NLR values than younger patients. In addition, we found that age had an impact on the correlation between immune parameters and PANSS scores.

Consistent with other studies in SCZ, we found increased NEU and NLR values in patients compared to healthy controls [18, 28, 29]. Our study provides further evidence that immune parameters, including NEU and NLR, were increased in young adults with SCZ compared with healthy controls. The increase in NEU and NLR may indicate a pro-inflammatory state of the immune system in young adult patients with SCZ, which is associated with the pathophysiology of SCZ. Our findings of increased NEU and NLR in young patients also indicate that these immune parameters can serve as potential biomarkers for SCZ, which may be helpful in diagnosis and treatment.

Furthermore, we found that NEU and NLR values were even higher in the older group than in the younger group, suggesting systemic inflammation depended on age in SCZ patients. This also indicates that younger patients have a different inflammatory profile compared to older patients. This finding was consistent with previous studies in the general population [30-32]. It is well-known that chronic, low-grade inflammation occurs with age and is associated with a variety of age-related diseases [33-35]. As the immune system ages, alterations in the immune system lead to a decrease in the production of new immune cells and an increase in the number of immune cells producing inflammatory cytokines. These findings support our finding that NEU and NLR values gradually increase with age in DNFES patients. Notably, the patients recruited in this study were young, with a mean age of 22.6 years, indicating that the DNFES patients in the early stage were prone to chronic, low inflammation. However, the exact causes of age-related inflammation in SCZ are not fully understood but are believed to involve a complex interplay of genetic, environmental, and lifestyle factors [36, 37]. Therefore, we cannot give a definitive explanation for these findings, which warranted elucidation in future studies.

Finally, we found that NEU count was positively associated with negative symptoms in the older group, whereas NLR values were positively associated with PANSS total score in the younger group. This finding suggests that age may have a moderate effect on the relationship between immune parameters and clinical symptoms in SCZ. NEU is the blood's most abundant type of WBC, accounting for approximately 50% to 70% of all WBCs. It plays an important role in the inflammatory response, recognizing and responding to inflammatory signals such as cytokines and chemokines. In addition to its direct defensive role, NEU can modulate the activity of other immune cells by releasing cytokines and other signaling molecules. Thus, higher NEU values in patients may suggest higher levels of pro-inflammatory cytokines and other immune mediators. Excessive proinflammatory cytokines lead to a downstream cascade of events contributing to oxidative stress and cellular dysfunction, which can affect many physiological processes [38]. We speculate that the increase in NEU counts and the associated downstream cascades of immune responses induced by unknown risk factors in the older patient group may be involved in the pathophysiology of negative symptoms. On the other hand, although LYM and NEU were not observed to correlate with symptoms in patients, the ratio between them (NLR) was identified as a more sensitive indicator for the symptoms in the younger patient group. Namely, NLR may be a more reflective indicator of the state of the immune system in the clinical manifestations of SCZ in younger patients. NLR is a biomarker of the balance between two immune pathways: the innate immune system and the adaptive immune system [39]. NLR has also been reported to be related to pro-inflammatory cytokines [40-43]. Our findings reveal early abnormalities in immune regulation in SCZ, which may be a potential target for SCZ treatment, especially when exploring therapeutic strategies targeting inflammatory pathways.

This study had four main limitations. First, the study's cross-sectional design captured only one time point, limiting the ability to measure changes in NLR, LYM, and NEU values over time. This design does not assess trends or track changes that may result from antipsychotics or the natural progression of SCZ. Second, this study measured a limited number of immune parameters. We did not measure other immune parameters. For example, pro-inflammatory cytokines, which play a key role in inflammation and immune responses in SCZ, were not measured. Third, this study did not assess other potential confounding factors that may influence immune activation and inflammation, such as stress, anxiety, depression, and sleep disorders. In particular, all participants recruited in our study were in the first episode of SCZ and were experiencing acute psychotic symptoms. Fourth, antipsychotic drugs, particularly second-generation antipsychotics (SGAs), have been found to have immunomodulatory effects. They may influence the immune system by altering the production of cytokines or affecting the activity of immune cells. Some of the patients recruited in our study had received antipsychotic medication for no more than 2 weeks previously.

## CONCLUSION

Overall, this study found higher NEU and NLR values in young patients with SCZ relative to healthy controls, and younger patients had higher NEU and NLR values than older patients. In addition, increased NEU and NLR correlated with symptoms in patients. This study provides new evidence for the role of immune parameters in a sub-group of young adult patients with SCZ. Further research in multiple disciplines is needed to understand better the link between immune parameters and SCZ in young patients and to elucidate the pathogenesis of SCZ.

## Figures and Tables

**Table 1:** Demographic and clinical characteristics in younger and older patients with schizophrenia (SCZ).

**Variable**	**Younger Group ** **(n=29)**	**Older Group ** **(n=35)**	**For *X^2^* (*p*-value)**
Sex (M/F)	15/14	20/15	0.2(0.67)
Age (years)	19.2 ± 0.9	25.5 ± 3.3	101.5(<0.001)
Education (y)	8.4 ± 1.9	8.8 ± 3.5	0.3(0.60)
BMI (kg/m^2^)	19.1 ± 2.6	20.5 ± 2.7	4.4(0.04)
Onset age (y)	18.5 ± 1.0	24.7 ± 3.4	88.0(<0.001)
Antipsychotics	-	-	0.5(0.49)
Yes	10	15	-
No	19	20	-
**Biomarkers**			
NEU count (10^9^/L)	4.0 ± 1.3	4.8 ± 1.7	5.1(0.03)
LYM count (10^9^/L)	2.2 ± 0.6	2.1 ± 0.8	0.5(0.47)
NLR	2.0 ± 1.2	2.6 ± 1.4	4.0(0.05)
**Clinical Symptoms**			
P subscore	19.4 ± 5.3	18.8 ± 4.5	0.04(0.85)
N subscore	18.9 ± 6.1	17.3 ± 8.3	0.8(0.37)
G subscore	31.0 ± 6.3	32.8 ± 7.1	1.1(0.30)
Total score	68.6 ± 11.0	68.7 ± 13.0	0.004(0.95)

**Abbreviations:** BMI Body mass index; NEU neutrophils; LYM lymphocyte; P positive symptoms; N negative symptoms; G general psychology; PANSS the Positive and Negative Syndrome Scale.

**Table 2:** Correlations between immune parameters and clinical symptoms in the younger and older groups.

**-**	**-**	**P Subscore**	**N Subscore**	**G Subscore**	**Total Score**
**Younger Group (n=29)**					
NLR	r	0.18	0.25	0.39	0.45
	p	0.36	0.19	0.04	0.01
LYM	r	-0.06	-0.34	-0.21	-0.33
	p	0.77	0.07	0.28	0.09
NEU	r	0.16	0.17	0.37	0.38
	p	0.41	0.39	0.05	0.04
**Older Group (n=35)**					
NLR	r	0.06	0.36	0.26	0.35
	p	0.75	0.03	0.13	0.04
LYM	r	0.04	-0.16	-0.24	-0.19
	p	0.82	0.36	0.17	0.27
NEU	r	0.13	0.51	0.24	0.44
	p	0.45	0.002	0.16	0.007

**Abbreviations:** NEU neutrophils; LYM lymphocyte; NLR neutrophils-to-lymphocyte.

## Data Availability

The data supporting the findings of this study are available on request from the corresponding author. The data are not publicly available due to privacy or ethical restrictions.

## LIST OF ABBREVIATIONS

ANOVA: Analysis of Variance
BMI: Body Mass Index
DNFES: Drug-naïve First-episode Schizophrenia
DSM-IV: Diagnostic and Statistical Manual of Mental Disorders, Fourth Edition
LYM: Lymphocyte
NEU: Neutrophil
NLR: Neutrophil-to-lymphocyte Ratio
PANSS: The Positive and Negative Syndrome Scale
SCID: Structured Clinical Interview for DSM Disorders
SCZ: Schizophrenia
SD: Standard Deviation
WBC: White Blood Cell
